# Impact of polyols on Oral microbiome of Estonian schoolchildren

**DOI:** 10.1186/s12903-019-0747-z

**Published:** 2019-04-18

**Authors:** Jelena Štšepetova, Jaak Truu, Riina Runnel, Rita Nõmmela, Mare Saag, Jana Olak, Hiie Nõlvak, Jens-Konrad Preem, Kristjan Oopkaup, Kaarel Krjutškov, Eino Honkala, Sisko Honkala, Kauko Mäkinen, Pirkko-Liisa Mäkinen, Tero Vahlberg, Joan Vermeiren, Douwina Bosscher, Peter de Cock, Reet Mändar

**Affiliations:** 1 Department of Microbiology, Institute of Biomedicine and Translational Medicine, University of Tartu, Tartu, Estonia; 2 Competence Centre on Health Technologies, Tartu, Estonia; 3 Institute of Dentistry, University of Tartu, Tartu, Estonia; 4 Faculty of Science and Technology, University of Tartu, Tartu, Estonia; 5 Estonian Genome Center, University of Tartu, Tartu, Estonia; 6 Institute of Clinical Dentistry, University of Tromso, Tromso, Norway; 7 Institute of Dentistry, University of Turku, Turku, Finland; 8 Faculty of Medicine, University of Turku, Turku, Finland; 9 Cargill R&D Centre Europe, Vilvoorde, Belgium

**Keywords:** Polyol, Erythritol, Oral microbiome, Saliva, qPCR, Next generation sequencing

## Abstract

**Background:**

Oral microbiome has significant impact on both oral and general health. Polyols have been promoted as sugar substitutes in prevention of oral diseases. We aimed to reveal the effect of candies containing erythritol, xylitol or control (sorbitol) on salivary microbiome.

**Methods:**

Ninety children (11.3 ± 0.6 years) consumed candies during 3 years. Microbial communities were profiled using Illumina HiSeq 2000 sequencing and real-time PCR.

**Results:**

The dominant phyla in saliva were *Firmicutes* (39.1%), *Proteobacteria* (26.1%), *Bacteroidetes* (14.7%), *Actinobacteria* (12%) and *Fusobacteria* (6%). The microbiome of erythritol group significantly differed from that of the other groups. Both erythritol and xylitol reduced the number of observed bacterial phylotypes in comparison to the control group. The relative abundance of the genera *Veillonella, Streptococcus* and *Fusobacterium* were higher while that of *Bergeyella* lower after erythritol intervention when comparing with control. The lowest prevalence of caries-related mutans streptococci corresponded with the lowest clinical caries markers in the erythritol group.

**Conclusions:**

Daily consumption of erythritol, xylitol or control candies has a specific influence on the salivary microbiome composition in schoolchildren. Erythritol is associated with the lowest prevalence of caries-related mutans streptococci and the lowest levels of clinical caries experience.

**Trial registration:**

ClinicalTrials.gov Identifier NCT01062633.

**Electronic supplementary material:**

The online version of this article (10.1186/s12903-019-0747-z) contains supplementary material, which is available to authorized users.

## Background

The oral microbiome is comprised of around 700 to 1000 microbial species according to the studies using high-throughput technology and has significant impact on both the oral and general health [1–3]. High diversity between individuals has been revealed, although a significant proportion of bacterial sequences of healthy individuals are identical [4–7].

The study of Ling et al. [8] has shown that the salivary microbiome in healthy children is more diverse compared to adults. Keijser et al. [2] have found that, in comparison to adults, children’s saliva appears to include a higher proportion of *Firmicutes* and *Actinobacteria* and a lower proportion of *Bacteroidetes* and *Fusobacteria*. Geographical differences have been noted as well: in comparison to healthy Americans, a higher relative abundance of *Bacteroides* (26.64%) and *Proteobacteria* (19.85%) and lower relative abundance of *Firmicutes* (38.53%) were found in the Netherlands [2].

Dental caries is associated with an increase in the proportion of acidogenic and aciduric bacteria, especially mutans streptococci. Although *Streptococcus sobrinus* is less frequently detected than *Streptococcus mutans*, several studies have reported its association with caries activity. In children, the caries increment with both *S. mutans* and *S. sobrinus* is higher than in those with *S. mutans* alone [9, 10]. At the same time the imbalance between the oral bacteria that involves suboptimal low levels of streptococci and high levels of anaerobic and Gram negative bacteria such as *Aggregatibacter actinomycetemcomitans* may lead to periodontitis [11, 12].

The microbiome in the oral cavity may be influenced by different factors such as oral hygiene, diet, antimicrobial and physicochemical factors [13]. Application of sugar alcohols (polyols) may be used for prevention of oral diseases to promote sugar substitutes in caries control. The most widely used sugar alcohols are sorbitol (a hexitol) and xylitol (a pentitol). Xylitol has been found to reduce the amount of plaque, the viability and survival of virulent *S. mutans* [14]. Erythritol (a tetritol) is a newer and therefore less used polyol, while studies suggest its caries-preventive effect [15–21]. Polyols resist fermentation and acidogenesis by the bacteria of dental plaque [22, 23] and are not absorbed via the stomach [24]. It has been recognized that regular use of polyol-containing chewing gums could play a role in preventing caries by increasing salivary flow through mastication, reversing decreases in plaque pH and enhancing remineralization of subsurface enamel lesions [25–28].

Previous studies of the polyols’ effect on oral microbiome have been focused on certain groups of bacteria and routinely performed by using of culture-based techniques. The influence of polyols on total salivary microbiome has not been described.

The aim of this study was to reveal the effect of erythritol, xylitol and sorbitol (control) candies on salivary microbiome after 3 years of a double-blind randomized controlled prospective clinical trial in Estonian schoolchildren. Two molecular approaches were combined, high-throughput sequencing that allows a global systemic view of the human oral microbiome, and real-time PCR with specific primers that provide an accurate and sensitive method for quantification of individual bacteria in total bacterial count [9, 29–31]. Further, associations were made between the changes in the salivary microbiome and clinical caries experience as previously described by Honkala et al. [15] and Falony et al. [17].

## Methods

### Study population

This study was part of the larger double-blind randomized controlled prospective clinical trial “Effect of erythritol and xylitol on dental caries prevention in children” (ClinicalTrials.gov Identifier NCT01062633) that was carried out at the University of Tartu from 2008 to 2011 and included 485 first- and second-grade schoolchildren from 10 randomly selected schools of south-eastern Estonia (10% of the all schools in this area) [15–17]. All school classes were randomly allocated into 3 intervention groups according to their consumption of erythritol, xylitol or sorbitol (control) candies. The subgroup for microbiological study comprised randomly selected 90 children (*n* = 30 from each group; 53 girls and 37 boys) with mean ± SD age 11.3 ± 0.6 years (range 10.2–12.5; quartiles 10.8–11.8) (Table 1). Subject recruitment, inclusion and exclusion criteria are more thoroughly described in our previous papers [15–17].

**Table 1:** Clinical parameters of the study subjects (*n* = 90) at the end of the intervention trial (mean ± SD)

Clinical data	Erythritol	Xylitol	Sorbitol (control)	*P* values
Age (y)	11.6 ± 0.62	11.3 ± 0.6	11.1 ± 0.63	*NS*
*DMFT*	2.06 ± 1.71 ^1^	3.06 ± 2.18	3.62 ± 2.55 ^1^	^1^ 0.013
*DMFS*	3.2 ± 3.63 ^2^	4.37 ± 3.42	5.58 ± 4.50 ^2^	^2^ 0.025

DMFT, number of decayed, missing and filled teeth; DMFS, number of decayed, missing and filled tooth surfaces

^1^*P* = 0.013 versus sorbitol

^2^*P* = 0.025 versus sorbitol

The study was performed according to Declaration of Helsinki and approved by the Ethics Review Committee on Human Research of the University of Tartu (no. 166/7 17.12.2007). The written agreement forms were signed by parents or caretakers of the participants.

### Clinical examination and sample collection

The clinical examination and saliva collection was made by four calibrated examiners and by four dental assistants in the clinic of the Department of Stomatology, Tartu University as described previously [14, 16, 17]. The children were informed not to brush their teeth in the morning of the examination day. Caries status was registered by ICDAS II coding system [32] for analyzing and converted into DMF indices where DMFT indicates number of decayed, missing and/or filled teeth (maximum 28), and DMFS number of decayed, missing and/or filled tooth surfaces (maximum 128) [33]. Salivary samples were collected during the last examination after 3 year consumption of polyols, during 2-min chewing of a piece of paraffin and stored at − 80 °C until analyzed by molecular methods.

### Intervention

All children were instructed on oral hygiene during the examination each year. The new toothbrush and fluoridated toothpaste (0.24% sodium fluoride) were given to children twice a year. Throughout the intervention trial (2008–2011), pupils consumed erythritol-, xylitol-, and sorbitol-containing candies. The candies’ consumption was supervised by their teachers and took place during 200 school days per year for 3 years in total. Each participant consumed four small candies three times per school day. Total daily intake of polyol was about 7.5 g. Candies were distributed before the start of the classes (8 a.m.), after school lunch (10:30 a.m.), and at the end of the school day (1:30 or 2:15 p.m.). Candies were not used during the weekends or holidays.

### Molecular methods

Salivary DNA was isolated applying the QiaAmp Blood Kit (Qiagen, Hilden, Germany). Real-time PCR was applied to quantify the indicator species *Streptococcus mutans, Streprococcus sobrinus,* and *Aggregatibacter actinomycetemcomitans.* Microbial communities were profiled using Illumina® HiSeq 2000 [36]. The details of molecular methods are presented in Additional file 1: Table S6.

### Statistical analyses

The statistical analysis of clinical and qPCR data was performed using SIGMASTAT 2.0 (Systat Software, Chicago, USA) statistic software package. According to the data descriptive statistics, Fisher exact test, Bonferroni correction and Mann-Whitney rank sum test were applied to compare the differences in microbiological indices. Spearman rank order correlation test was used to test the associations between microbiological and clinical data. All differences were considered statistically significant if *P* < 0.05.

In case of principal component (PCA) analysis, only 63 OTUs were retained in the data set. The criterion was that OTU had to be present at least in one sample with relative abundance bigger than 1%. Relative abundance values of phylotypes (OTUs) were Hellinger transformed prior applying principal component analysis. The effect of intervention on bacterial community structure was evaluated applying one-way permutational multivariate analysis (PERMANOVA) followed by between group analysis [45]. Before PERMANOVA, the distance-based test for homogeneity of multivariate dispersions was done. One-way ANOVA was applied to assess differences in diversity indices between study groups. Zero-inflated Gaussian mixture model was applied to detect differentially abundant OTUs and genera between intervention groups using metagenomeSeq software [46].

## Results

### Clinical indices

The effect of polyols on dental caries indices has been evaluated after 3 years consumption of erythritol, xylitol or sorbitol (control). At the end of the trial, the values of DMFT and DMFS in the subgroup of 90 schoolchildren were significantly lower in the erythritol group versus the sorbitol group (Table 1). Additional clinical data of the full cohort are presented elsewhere [15–17]. The data of the subgroup for the microbiological study may differ somewhat from that of the whole study group since they are based on the random sample of the whole data.

### Analysis of the mock community for Illumina sequencing

In order to check sequencing quality, the mock (artificial) community was analyzed along with all clinical samples. Mock community consisted of seven bacterial strains commonly found in the human oral cavity. Results of mock community analysis are presented in Additional file 1: Table S2. All strains were recovered and identified from the obtained data set.

### Salivary microbiome composition

**Fig. 1:**
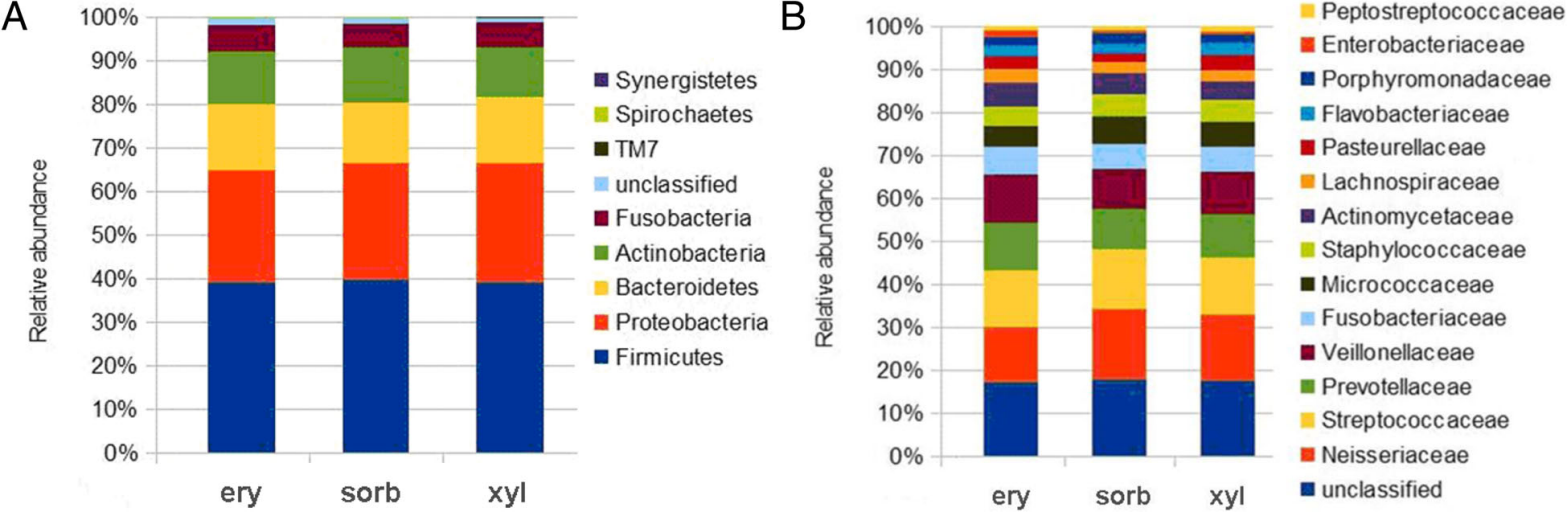
Relative abundance of bacterial phyla (**a**) and families (**b**) within studied groups. Groups: ery – erythritol, sorb – sorbitol, xyl – xylitol

**Fig. 2:**
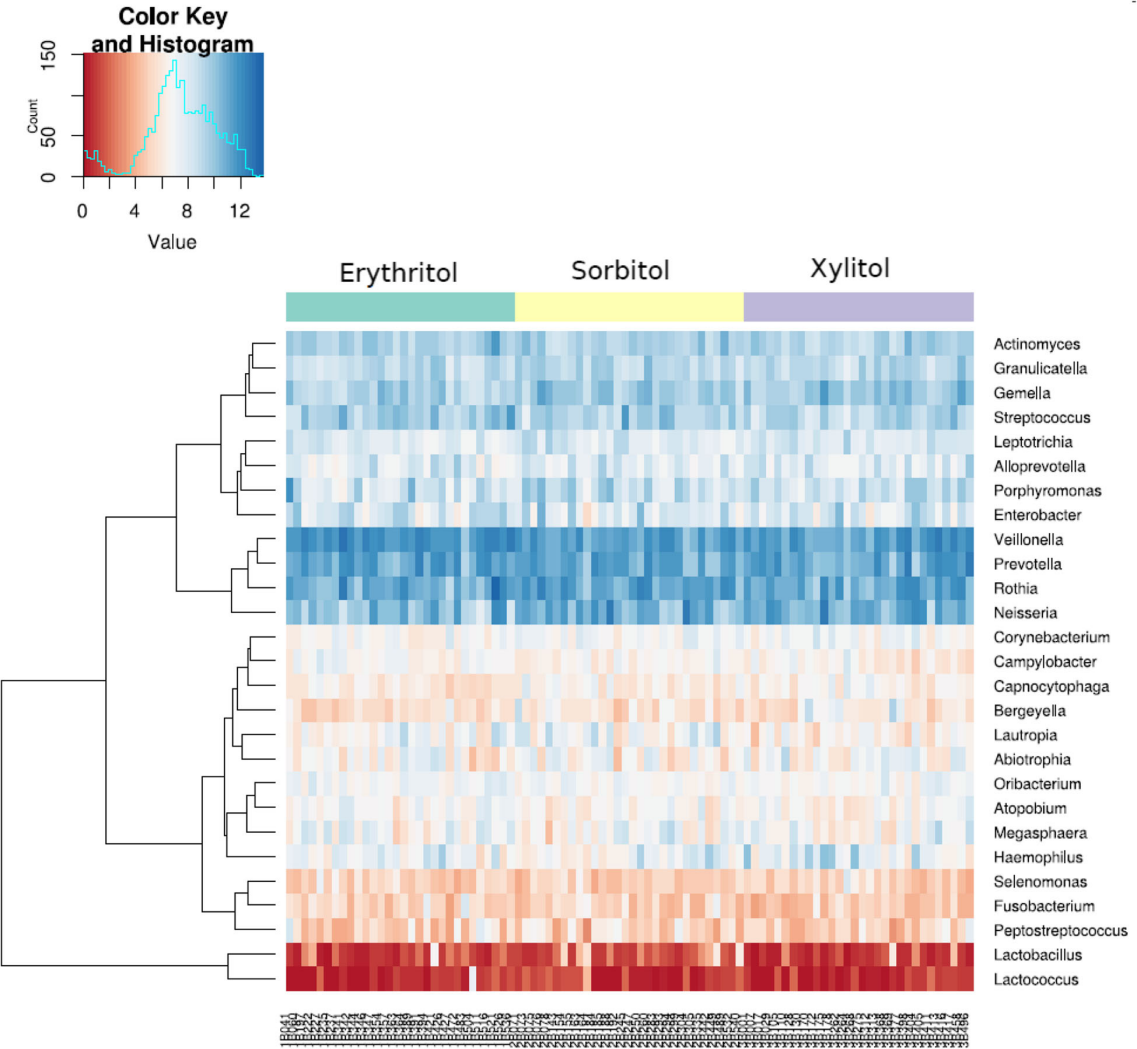
Heat map showing the relative abundance of the most predominant genera in saliva. Row represents the relative percentage of each bacterial genus, and column stands for different samples

The five dominant phyla (among all obtained sequences) were *Firmicutes* (39%), *Proteobacteria* (26%), *Bacteroidetes* (15%), *Actinobacteri*a (12%) and *Fusobacteria* (6%) according to RDP classifier. Among the identified 16 different bacterial families the most prevalent were *Neisseriaceae*, *Streptococcaeceae*, *Prevotellaceae* and *Veillonellaceae* across all intervention groups. The proportions of unclassified phylum and family level sequences were 2 and 18%, respectively. Relative abundance of bacterial phyla and families in the groups are shown on Fig. 1 (a, b). The most abundant phylotypes belonged to Gram negative cocci *Veillonella* sp. and *Neisseria* sp.*,* Gram negative anaerobic rod *Prevotella* sp*.,* and Gram positive cocci *Rothia* sp. and *Gemella* sp. (Table 2)*.* Similar results was found when analysing the data on genus level (Fig. 2).

**Table 2:** Proportions of ten most abundant bacterial phylotypes (more than 1%) in saliva microbiomes (mean ± SD)

OTU	Identification Greengenes	Identification HOMD 16S RefSeq	Erythritol	Xylitol	Sorbitol
Otu037	Unclassified *Veillonellaceae*	*Veillonella parvula*	12.4 ± 3.4	9.4 ± 3.1	7.8 ± 2.8
Otu005	*Veillonella* sp.	*Veillonella* sp.	10.8 ± 3.4	7.9 ± 2.8	8.4 ± 3.5
Otu068	*Rothia mucilaginosa*	*Rothia mucilaginosa*	7.4 ± 4.1	7.4 ± 2.7	7.9 ± 3.7
Otu070	*Prevotella* sp.	*Prevotella* sp.	6.5 ± 3.1	5.9 ± 3.2	6.3 ± 3.2
Otu038	*Rothia mucilaginosa*	*Rothia mucilaginosa*	5.7 ± 2.7	5.5 ± 2	7.4 ± 2.8
Otu009	*Prevotella* sp.	*Prevotell*a sp.	6.4 ± 3.5	6.7 ± 3.6	5.2 ± 2.2
Otu015	*Neisseria* sp.	*Neisseria* sp.	3.6 ± 2.2	5.5 ± 2.6	6.6 ± 4.9
Otu018	Unclassified *Neisseriaceae*	*Neisseria* sp.	2.1 ± 2.2	4.2 ± 3.5	4 ± 3.7
Otu096	*Gemella sanguinis*	*Gemella morbillorum*	2.3 ± 1.2	3.7 ± 1.9	3 ± 1.3
Otu209	Unclassified *Neisseriaceae*	*Neisseria* sp.	2.2 ± 1.6	3.3 ± 2.6	2.4 ± 2.2

### Influence of polyols on the diversity and structure of saliva microbiome

The intervention groups were different from each other (PERMANOVA, *P* < 0.001) and the erythritol group was different from the xylitol and sorbitol groups (pair-wise a posteriori comparisons, *P* < 0.001). Both erythritol and xylitol reduced the number of observed bacterial phylotypes in saliva in comparison to the sorbitol group (ANOVA, *P* < 0.001, Table 3) The inverted Simpson index, indicator of the community diversity was not significantly different between the test and sorbitol groups (ANOVA, *P* = 0.075). These results indicated that, compared to the sorbitol, in saliva samples there are less OTUs with high relative abundance after intervention with erythritol and xylitol.

**Table 3:** Average species richness and inverted Simpson index values (mean ± SD)

Treatment groups	Species richness (number of OTUs) ^1^	Inverted Simpson Index ^2^
Erythritol	310 ± 18	17.4 ± 4.9
Xylitol	301 ± 21	19.0 ± 3.8
Sorbitol	327 ± 20	20.3 ± 6.1

^1^*P* < 0.001 (one-way ANOVA)

^2^*P* = 0.075 (one-way ANOVA)

**Fig. 3:**
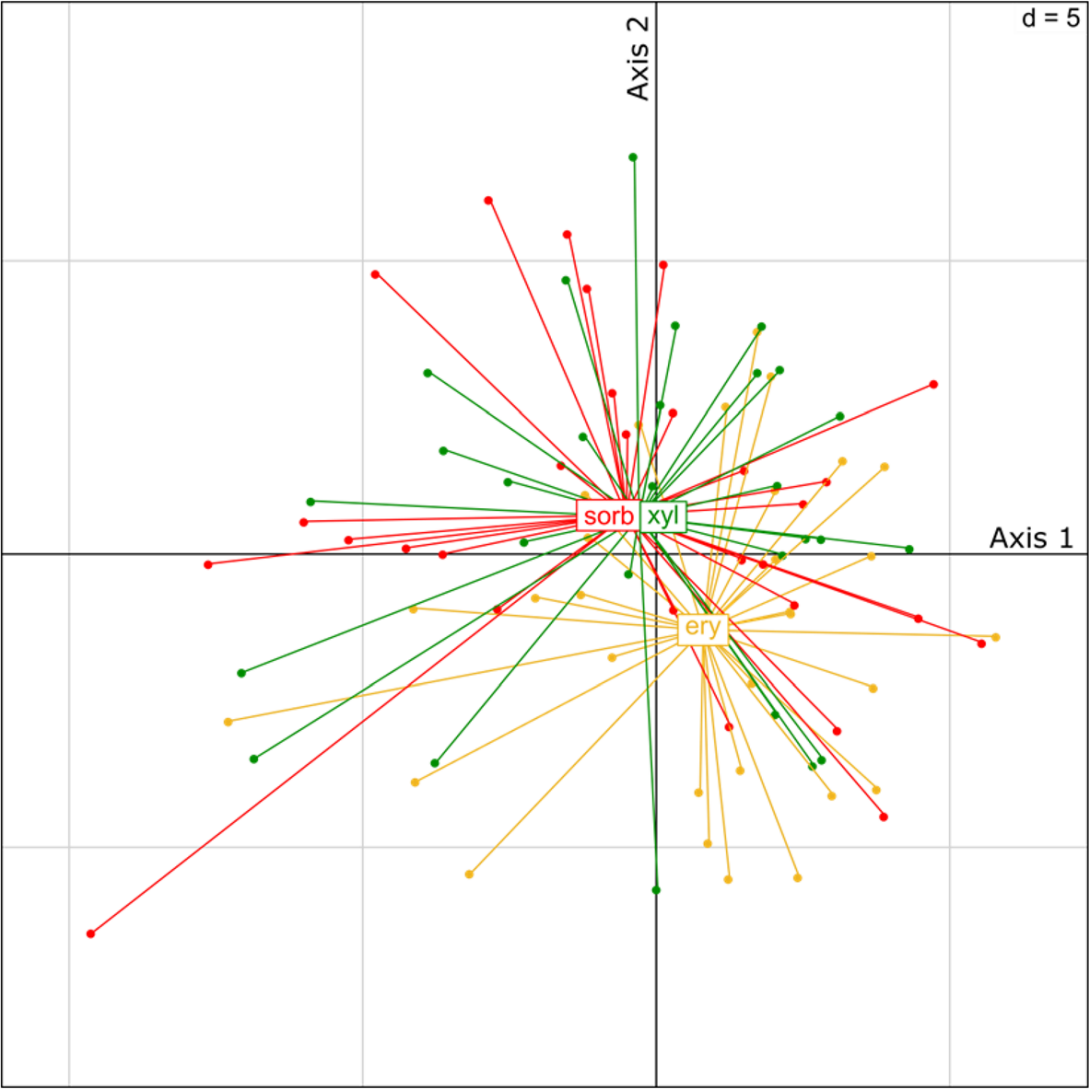
Plot of principal component analysis based on Hellinger transformed OTU relative abundance values. Individual saliva microbiome samples are connected to treatment centroids. First and second principal axes describe 16.7 and 13.3% of overall variation, respectively. Abbreviations: ery - erythritol, sorb - sorbitol, xyl - xylitol

Principal component analysis (PCA) was applied to visualize the differences in the microbial communities of saliva samples. The centroid of the erythritol group samples is more distant from the group centroids of other two treatments on PCA plot which is in concordance of the PERMANOVA analysis results (Fig. 3).

Analysis of differentially abundant OTUs showed that erythritol intervention increased the relative abundance of genus *Veillonella* and *Streptococcus* members in saliva compared to both sorbitol and xylitol groups (Additional file 1: Tables S3 and S4). Relative abundance of genus *Prevotella* and *Oribacterium* increased in the erythritol group compared to sorbitol, too, while no clear difference appeared in comparison with xylitol group. At the same time the relative abundance of *Gemella* and *Neisseria* decreased in case of erythritol in comparison to sorbitol. The relative abundance of *Rothia, Actinomyces, Enterobacter* and *Solobacterium* was higher while that of *Leptotrichia* and *Alloprevotella* lower for erythritol in comparison to xylitol.

Similar analysis was performed also on the genus level (Table 4 and Additional file 1: Table S5). This analysis confirmed a significantly higher relative abundance of genus *Veillonella* and *Streptococcus* members in saliva in erythritol group compared to the xylitol and sorbitol group. In addition, higher abundance of *Fusobacterium* while a lower abundance of *Bergeyella* was noted in erythritol group in comparison with xylitol group.

**Table 4:** Differentially abundant genera between erythritol and sorbitol groups, and erythritol and xylitol groups based zero-inflated Gaussian mixture model

Genus	Erythritol compared to sorbitol logFC	Erythritol compared to xylitol logFC
*Bergeyella*	0,47	**0,83** (*P* < 0.05)
*Fusobacterium*	−0,34	**−0,93** (*P* < 0.01)
*Streptococcus*	−0,36	**−0,69** (*P* < 0.0001)
*Veillonella*	**−0,60** (P < 0.001)	−0,50

A negative value for fold change (logFC) indicates an increase of in the relative abundance of a particular genus in the erythritol group compared to the sorbitol or xylitol group. Statistically significant fold changes are shown in bold and followed by *P* value in parenthesis

### Prevalence and proportions of indicator bacteria in saliva samples according to real-time PCR

The prevalence and counts of total and three indicator bacteria in saliva samples according to real-time PCR are presented in Table 5. *S. sobrinus* displayed the lowest prevalence in the erythritol group but also in the xylitol group its prevalence was significantly lower compared to the sorbitol (*P* = 0.015 and *P* = 0.011, respectively, versus sorbitol). The counts of *S. mutans* and *S. sobrinus* were significantly positively correlated (r_s_ = 0.265, *P* = 0.0035). Neither the prevalence nor counts of *A. actinomycetemcomitans* between the groups was statistically significant.

**Table 5:** Real-time PCR results: counts (log_10_ plasmid copies/ml saliva; mean ± SD) and prevalence (%) of total bacteria and three indicator bacteria

Bacteria spp.	Erythritol		Xylitol		Sorbitol	
	plasmid copies/ml saliva	%	plasmid copies/ml saliva	%	plasmid copies/ml saliva	%
Total bacteria	9.8 ± 0.4	100	9.94 ± 0.3	100	9.8 ± 0.3	100
*S. mutans*	3.3 ± 1.6	83	3.6 ± 1.5	87	3.6 ± 0.96	97
*S. sobrinus*	0.59 ± 1.4	17 ^1^	0.72 ± 1.5	20 ^2^	1.17 ± 1.6	37 ^1, 2^
*A. actinomycetem-comitans*	1.17 ± 1.8	33	1.10 ± 2.0	27	1.35 ± 2.1	30

^1^*P* = 0.015 versus sorbitol; ^2^ *P* = 0.011 versus sorbitol

### Associations between microbiological and clinical data

We revealed some correlations between bacteria and caries indices. The relative abundance of OTU022 and OTU257 (*Leptotrichia*) was lower in the erythritol group and positively associated with DMFT (R = 0.22, *p* = 0.04 and R = 0.21, p = 0.04, respectively). The relative abundance of OTU034 (*Enterobacter*) was higher in the erythritol group and negatively associated with DMFT (R = − 0.28, *p* = 0.008) (data not shown). The correlation analysis on genus level confirmed negative association between *Enterobacter* and caries indicators and revealed positive association between caries indicators and bacterial genera *Lactobacillus, Bergeyella* and *Capnocytophaga* (Table 6).

**Table 6:** Spearman’s rank-order correlation between clinical and microbiological data. Results presented as correlation coefficient r_s_; *p* value

Clinical data	Bacteria spp.	Presence of bacteria (r_s_; p)	Counts of bacteria (r_s_; p)
*DMFT*	Total bacteria	NS	NS
	*S. mutans*	0.271; 0.004	0.308; 0.001
	*S. sobrinus*	NS	NS
	*A. actinomycetemcomitans*	NS	NS
	*Enterobacter sp.*		−0.28; 0.010
	*Lactobacillus sp.*		0.27; 0.012
*DMFS*	Total bacteria	NS	NS
	*S. mutans*	0.248; 0.009	0.306; 0.001
	*S. sobrinus*	NS	NS
	*A. actinomycetemcomitans*	NS	NS
	*Bergeyella sp.*		0.21; 0.049
	*Capnocytophaga sp.*		0.23; 0.039
	*Enterobacter sp.*		−0.30; 0.005
	*Lactobacillus sp*		0.34; 0.001

NS – not significant

Total bacteria, *S. mutans, S. sobrinus* and *A. actinomycetemcomitans* were detected using qPCR method while other bacteria using high-throughput sequencing

The correlation analysis between clinical caries experience indices and three indicator bacteria (*S. mutans*, *S. sobrinus* and *A. actinomycetemcomitans*) revealed positive associations between caries indices and *S. mutans* (Table 6).

## Discussion

Our study revealed that the four dominant phyla in the salivary microbiome of schoolchildren, independently of the type of polyol consumed, were *Firmicutes*, *Proteobacteria*, *Bacteroidetes* and *Actinobacteria* while the most prevalent families were *Neisseriaceae*, *Streptococcaceae*, *Staphylococcaceae* and *Veillonellaceae*. However, after 3-year consumption of erythritol, xylitol or sorbitol clear differences were shown in the salivary microbiome. Both erythritol and xylitol showed lower numbers of observed bacterial phylotypes in comparison to the sorbitol group. According to PERMANOVA analysis the microbiome of the erythritol group differed significantly from that of the xylitol and sorbitol group. The erythritol intervention showed a higher relative abundance of *Veillonella, Streptococcus and Fusobacterium* but a lower relative abundance of *Bergeyella.* The prevalence of caries-related mutans streptococci was also the lowest in the erythritol group that corresponded with the lowest clinical caries markers in this group. To our knowledge this is the first study revealing the effect of different polyols on the salivary microbiome and their association with oral health applying high-throughput sequencing.

Sugar alcohols are noncyclic hydrogenated carbohydrates which not only offer a wide range of sweetness and cooling effect, but also non-cariogenic and less calorigenic properties. Due to their health-promoting benefits they are emerging food ingredients [47]. Previous studies have shown that xylitol, a pentitol type sugar alcohol, can be used as a safe and effective caries-limiting sweetener. It reduces the growth of dental plaque, interferes with the growth of caries-associated bacteria, decreases the incidence of dental caries, and promotes remineralization of caries lesions [19, 48, 49]. This was supported by our study where the children consumed 7.5 g of xylitol per day and the prevalence of mutans streptococci, especially *S. sobrinus*, was lower in their saliva than in the children consuming control candies. Erythritol, a tetritol-type alditol, is another though less investigated caries-reducing polyol. Unlike sorbitol and xylitol, erythritol is well tolerated, rapidly and almost completely absorbed from the small intestine, not metabolized and excreted unchanged in the urine thereby being almost non-caloric [50, 51]. Erythritol has been shown to significantly reduce the dental plaque weight [16, 18] as well the development of enamel/dental caries [15, 17]. Erythritol reduces growth of the plaque-related biofilm, and streptococci do not produce neither lactic nor other acids from erythritol [16, 18, 19, 52–54].

Recent advances in molecular microbiological techniques have allowed comprehensive surveys of complex bacterial communities, including the microbiota in the oral cavity that is comprised of nearly 700 to 1000 microbial species. There are two types of surfaces in the oral cavity that bacteria can colonize: the hard surfaces of teeth and the soft tissue of the oral mucosa. The microbial population in the saliva originates from both these surfaces and is therefore a mixture of the different microbial consortia [55–57]. The dominant phyla identified in the children’s salivary microbiota upon 3-year polyol consumption (*Firmicutes*, *Proteobacteria*, *Bacteroidetes, Actinobacteria, Fusobacteria)* were similar to the dominant phyla identified in observational non-intervention studies [2, 8, 58] that is in alignment with our findings showing that the polyols induced changes on genus level but not phylum level. Illumina technology also revealed *Streptococcus* and *Neisseria* [59] as most prevalent genera in the oral microbiome which corresponds partly with our data identifying *Neisseria, Veillonella, Prevotella* and *Rothia,* followed by *Streptococcus* and *Gemella* as most prevalent genera.

PCA analysis revealed that impact of xylitol on bacterial community is rather similar to the sorbitol, while in the case of erythritol the changes in community structure were clearly visible. The abundance of certain genera (*Veillonella, Streptococcus, Fusobacterium*) was higher while that of the others was lower (*Bergeyella*) after erythritol intervention compared to the sorbitol group. The higher abundance of *Veillonella* in the erythritol group is an intriguing finding since the available data concerning this genus are quite conflicting. Recent metagenomic studies have revealed this microorganism among the most predominant microorganisms in the saliva of healthy individuals [60]. In some studies *Veillonella* sp. has been related to caries [61, 62] but in the other studies it has been less frequently found in patients with caries [63] which corresponds to our data. Interestingly, co-cultures of *Veillonella* with *S. mutans* have been shown to produce more acid than any one of these species separately [64], suggesting that synergistic effects take place. A lack of this synergy because of the low prevalence of mutans streptococci in the erythritol group could potentially explain the lower acid concentrations found in the dental plaque of these children [16]. In another recent polyol study where the oral bacteria were tested using microarray method, no changes in the salivary microbiota took place in the xylitol group while *Veillonella atypica* showed a significant decrease in the sorbitol group [65], the latter being in accordance with our data.

The genus *Streptococcus* was more abundant in erythritol group, too. Oral streptococci are highly heterogenic and they are divided into five different groups: Mutans group (prominent members are *Streptococcus mutans* and *Streptococcus sobrinus*), Salivarius group (*Streptococcus salivarius*), Anginosus group (*Streptococcus anginosus* and *Streptococcus intermedius*), Sanguinis group (*Streptococcus sanguinis* and *Streptococcus gordonii*), and Mitis group (*Streptococcus mitis* and *Streptococcus oralis*) [66]. According to the HOMD database the OTU275 was close to oral taxon 423 that is non-cariogenic *Streptococcus mitis,* thus, the increase of this taxon can be considered as a favorable shift.

Relative abundance of *Fusobacteria* was quite low (less than 3%) in our study population after polyol consumption. These bacteria co-aggregate with most other oral bacteria that are important bridging organisms between early and late colonizers during plaque formation. Some members of this phylum*,* such as *Leptotrichia* can co-aggregate with potential cariogenic bacteria and may significantly associate with dental caries [67]. In accordance, our study showed a positive association between the relative abundance of *Leptotrichia* and DMFT which decreased after erythritol intervention. Little is known about difficult-to-culture oral bacterium *Bergeyell*a. It has been found from intact enamel surfaces of the children with caries [68] but it has also been associated with extra-oral infections and pregnancy complications [69].

In our study, quantitative real-time polymerase chain reaction was additionally used for rapid and accurate quantification of common oral pathogens like caries-associated *S. mutans* and *S. sobrinus,* and periodontitis-associated *A. actinomycetemcomitans* [9, 70]. qPCR assay has very large dynamic range of target molecule determination because real-time PCR products allows to quantify the amplified products in the log phase of reaction and the overall structure of bacterial communities formed within various oral sites has been revealed [9]. Our data revealed clear association of erythritol intervention with reduced prevalence of *S. sobrinus* while reduction of *S. mutans* was slightly above significance level. Both species have been significantly associated with dental caries, general prevalence of *S. mutans* being higher than that of *S. sobrinus* while the latter has been associated with more aggressive caries in children [71]. In our substudy also the clinical caries experience markers (DMFT, DMFS) displayed the lowest values in the erythritol group. It has been suggested that an ideal polyol would reduce the counts of the aciduric bacteria without simultaneous increase in counts of periodontal pathogens. In our study neither prevalence nor counts of periodontitis-associated *A. actinomycetemcomitans* were different between the groups.

As a limitation, periodontal status was not investigated in these children since the periodontal diseases are very infrequent in this age. At the same time, the counts of periodontitis-associated *A. actinomycetemcomitans* were detected in our subjects but they did not display any deviations from the generally low proportion.

## Conclusions

Daily erythritol consumption showed differentiating effects on the salivary microbiome composition in schoolchildren when compared to xylitol or sorbitol (control), while the impact of xylitol and sorbitol on the bacterial community was similar. Erythritol was associated with the lowest prevalence of caries-related mutans streptococci that corresponded to the lowest levels of clinical caries experience markers. This is the first study revealing the effect of polyols on the salivary microbiome and their association with oral health applying Illumina sequencing.

## Additional files

Additional file 1: **Table S1.** Specific primers and probes used for real-time PCR and Illumina HiSeq sequencing (V6 hypervariable region of the 16S rRNA gene). **Table S2.** Mock community analysis results. The initial composition of mock community (strains), phylotype identifications according to Greengenes (GG) and HOMD reference databases, and relative abundance of each phylotype are presented. **Table S3.** Differentially abundant OTUs between erythritol and control groups based zero-inflated Gaussian mixture model. A negative value for fold change (logFC) indicates an increase in the relative abundance of a particular OTU in the erythritol group compared to the control group. **Table S4.** Differentially abundant OTUs between erythritol and xylitol groups based zero-inflated Gaussian mixture model. A negative value for fold change (logFC) indicates an increase in the relative abundance of a particular OTU in the erythritol group compared to the xylitol group. **Table S5.** Differentially abundant genera between erythritol and sorbitol groups, and erythritol and xylitol groups based zero-inflated Gaussian mixture model. A negative value for fold change (logFC) indicates an increase of the relative abundance of a particular genus in the erythritol group compared to the sorbitol or xylitol group. Statistically significant changes are asterisked. **Table S6.** Details of molecular methods [34, 35, 37–44] (DOC 114 kb)

## Abbreviations

DMFS: Decayed, missing and/or filled tooth surfaces
DMFT: Decayed, missing and/or filled teeth
DNA: Deoxyribonucleic acid
ICDAS: International caries detection and assessment system
OTU: Operational taxonomic unit
PBS: Phosphate buffered saline
PCA: Principal component analysis
qPCR: Quantitative polymerase chain reaction
