# Supplementary material for: Impact of polyols on Oral microbiome of Estonian schoolchildren
Source: BMC Oral Health. 2019 Apr 18;19:60. doi: 10.1186/s12903-019-0747-z (PMC6471963; doi:10.1186/s12903-019-0747-z)
Supplement: Supplementary file 1 — Table S1. Specific primers and probes used for real-time PCR and Illumina HiSeq sequencing (V6 hypervariable region of the 16S rRNA gene). Table S2. Mock community analysis results. The initial composition of mock community (strains), phylotype identifications according to Greengenes (GG) and HOMD reference databases, and relative abundance of each phylotype are presented. Table S3. Differentially abundant OTUs between erythritol and control groups based zero-inflated Gaussian mixture model. A negative value for fold change (logFC) indicates an increase in the relative abundance of a particular OTU in the erythritol group compared to the control group. Table S4. Differentially abundant OTUs between erythritol and xylitol groups based zero-inflated Gaussian mixture model. A negative value for fold change (logFC) indicates an increase in the relative abundance of a particular OTU in the erythritol group compared to the xylitol group. Table S5. Differentially abundant genera between erythritol and sorbitol groups, and erythritol and xylitol groups based zero-inflated Gaussian mixture model. A negative value for fold change (logFC) indicates an increase of the relative abundance of a particular genus in the erythritol group compared to the sorbitol or xylitol group. Statistically significant changes are asterisked. Table S6. Details of molecular methods [34, 35, 37–44] (DOC 114 kb) [file 12903_2019_747_MOESM1_ESM.doc]

**Additional file 1**

**Table S1.** Specific primers and probes used for real-time PCR and Illumina HiSeq sequencing (V6 hypervariable region of the 16s rRNA gene).

| **Primer and probe** | **Sequence (5’-3’)** |
| --- | --- |
| **Total bacteria**  Forward  Reverse  Probe  ***S. mutans***  Forward  Reverse  Probe  ***S. sobrinus***  Forward  Reverse  Probe  ***A. actinomycetemcomitans***  Forward  Reverse  Probe  **V6 hypervariable region 16S**  Forward  Reverse | TGGAGCATGTGGTTTAATTCGA  TGCGGGACTTAACCCAACA  CACGAGCTGACGACA(AG)CCATGCA  GCCTACAGCTCAGAGATGCTATTCT  GCCATACACCACTCATGAATTGA  TGGAAATGACGGTCGCCGTTATGAA  TTCAAAGCCAAGACCAAGCTAGT  CCAGCCTGAGATTCAGCTTGT  CCTGCTCCAGCGACAAAGGCAGC  CTTACCTACTCTTGACATCCGAA  ATGCAGCACCTGTCTCAAAGC  AGAACTCAGAGATGGGTTTGTGCCTTAG  GAACGCGARGAACCTTACC  ACAACACGAGCTGACGAC |

**Table S2.** Mock community analysis results. The initial composition of mock community (strains), phylotype identifications according to Greengenes (GG) and HOMD reference databases, and relative abundance of each phylotype are presented.

| **OTU** | **Strains** | **Identification**  **Greengenes** | **Identification**  **HOMD 16S**  **RefSeq** | **Relative**  **abundance**  **(%)** |
| --- | --- | --- | --- | --- |
| 1 | *Aggregatibacter actinomycetemcomitans* | *A. actinomycetem-comitans* | *A. actinomycetem-comitans* | 19.3 |
| 2 | *Lactobacillus crispatus* | *L. crispatus* | *L. crispatus* | 17.5 |
| 3 | *Streptococcus sobrinus* | *Streptococcus sp.* | *S. sobrinus* | 16.6 |
| 4 | *Prevotella disiens* | *Prevotella sp.* | *Prevotella sp.* | 13.5 |
| 5 | *Lactobacillus gasseri* | *Lactobacillus* sp. | *L. gasseri* | 13.2 |
| 6 | *Streptococcus mutans* | *S. mutans* | *S. mutans* | 11.4 |
| 7 | *Lactobacillus jensenii* | *Lactobacillus sp.* | *L. jensenii* | 5.6 |

**Table S3**. Differentially abundant OTUs between **erythritol and control** groups based zero-inflated Gaussian mixture model. A negative value for fold change (logFC) indicates an increase in the relative abundance of a particular OTU in the erythritol group compared to the control group.

| **OTU** | **logFC** | **Phylum** | **Class** | **Order** | **Family** | **Genus** |  |  |
| --- | --- | --- | --- | --- | --- | --- | --- | --- |
| Otu001 | 0,53 | *Firmicutes* | *Bacilli* | *Bacillales* | *Staphylococcaceae* | *Gemella* |  |  |
| Otu005 | -0,51 | *Firmicutes* | *Clostridia* | *Clostridiales* | *Veillonellaceae* | *Veillonella* |  |  |
| Otu015 | 0,81 | *Proteobacteria* | *Betaproteo-bacteria* | *Neisseriales* | *Neisseriaceae* | *Neisseria* |  |  |
| Otu037 | -0,82 | *Firmicutes* | *Clostridia* | *Clostridiales* | *Veillonellaceae* | *Veillonella* |  |  |
| Otu275 | -0,91 | *Firmicutes* | *Bacilli* | *Lactobacillales* | *Streptococcaceae* | *Streptococcus* |  |  |
| Otu350 | -0,56 | *Firmicutes* | *Clostridia* | *Clostridiales* | *Lachnospiraceae* [XIVa] | *Oribacterium* |  |  |
| Otu387 | -0,92 | *Bacteroidetes* | *Bacteroides* | *Bacteroidales* | *Prevotellaceae* | *Prevotella* |  |  |

**Table S4**. Differentially abundant OTUs between **erythritol and xylitol** groups based zero-inflated Gaussian mixture model. A negative value for fold change (logFC) indicates an increase in the relative abundance of a particular OTU in the erythritol group compared to the xylitol group.

| **OTU** | **logFC** | **Phylum** | **Class** | **Order** | **Family** | **Genus** |  |  |  |
| --- | --- | --- | --- | --- | --- | --- | --- | --- | --- |
| Otu005 | -0,81 | *Firmicutes* | *Clostridia* | *Clostridiales* | *Veillonellaceae* | *Veillonella* |  |  |  |
| Otu006 | -0,88 | *Firmicutes* | *Bacilli* | *Lactobacillales* | *Streptococcaceae* | *Streptococcus* |  |  |  |
| Otu010 | 0,54 | *Firmicutes* | *Clostridia* | *Clostridiales* | *Lachnospiraceae* [XIVa] | *Oribacterium* |  |  |  |
| Otu022 * | 1,08 | *Fusobacteria* | *Fusobacteria* | *Fusobacteriales* | *Leptotrichiaceae* | *Leptotrichia* |  |  |  |
| Otu034 # | -0,82 | *Proteobacteria* | *Gammaproteo-bacteria* | *Enterobacteriales* | *Enterobacteriaceae* | *Enterobacter* |  |  |  |
| Otu037 | -0,79 | *Firmicutes* | *Clostridia* | *Clostridiales* | *Veillonellaceae* | *Veillonella* |  |  |  |
| Otu038 | -0,61 | *Actinobacteria* | *Actinobacteria* | *Actinomycetales* | *Micrococcaceae* | *Rothia* |  |  |  |
| Otu053 | -0,89 | *Firmicutes* | *Bacilli* | *Lactobacillales* | *Streptococcaceae* | *Streptococcus* |  |  |  |
| Otu063 | -0,78 | *Actinobacteria* | *Actinobacteria* | *Actinomycetales* | *Actinomycetaceae* | *Actinomyces* |  |  |  |
| Otu070 | -0,73 | *Bacteroidetes* | *Bacteroides* | *Bacteroidales* | *Prevotellaceae* | *Prevotella* |  |  |  |
| Otu074 | -0,80 | *Actinobacteria* | *Actinobacteria* | *Actinomycetales* | *Actinomycetaceae* | *Actinomyces* |  |  |  |
| Otu116 | 1,45 | *Bacteroidetes* | *Bacteroides* | *Bacteroidales* | *Prevotellaceae* | *Alloprevotella* |  |  |  |
| Otu134 | -0,55 | *Firmicutes* | *Erysipelotrichi* | *Erysipelotrichales* | *Erysipelotrichaceae* | *Solobacterium* |  |  |  |
| Otu199 | 0,87 | *Bacteroidetes* | *Bacteroides* | *Bacteroidales* | *Prevotellaceae* | *Prevotella* |  |  |  |
| Otu257 * | 1,53 | *Fusobacteria* | *Fusobacteria* | *Fusobacteriales* | *Leptotrichiaceae* | *Leptotrichia* |  |  |  |
| Otu282 | 1,09 | *Bacteroidetes* | *Bacteroides* | *Bacteroidales* | *Prevotellaceae* | *Prevotella* |  |  |  |
| Otu350 | -0,79 | *Firmicutes* | *Clostridia* | *Clostridiales* | *Lachnospiraceae* [XIVa] | *Oribacterium* |  |  |  |
| Otu387 | -1,09 | *Bacteroidetes* | *Bacteroides* | *Bacteroidales* | *Prevotellaceae* | *Prevotella* |  |  |  |

* The relative abundance of OTU022 and OTU257 (*Leptotrichia*) was lower in the erythritol group and positively associated with DMFT (r_s_=0.22, p=0.04 and r_s_=0.21, p=0.04, respectively).

# The relative abundance of OTU034 (*Enterobacter*) that were increased in the erythritol group were negatively associated with DMFT (r_s_= -0.28, p=0.008).

**Table S5.** Differentially abundant genera between erythritol and sorbitol groups, and erythritol and xylitol groups based zero-inflated Gaussian mixture model. A negative value for fold change (logFC) indicates an increase of the relative abundance of a particular genus in the erythritol group compared to the sorbitol or xylitol group. Statistically significant changes are asterisked.

| **Genus** | **Erythritol compared to sorbitol**  **logFC** | **Erythritol compared to xylitol**  **logFC** |
| --- | --- | --- |
| *Actinomyces* | -0,14 | -0,22 |
| *Alloprevotella* | 0,01 | 0,06 |
| *Atopobium* | -0,32 | -0,30 |
| *Bergeyella* | 0,47 | 0,83 * |
| *Campylobacter* | -0,33 | -0,50 |
| *Capnocytophaga* | 0,29 | 0,37 |
| *Corynebacterium* | 0,44 | 0,41 |
| *Enterobacter* | -0,40 | -0,25 |
| *Fusobacterium* | -0,34 | -0,93 * |
| *Granulicatella* | 0,27 | 0,07 |
| *Haemophilus* | -0,18 | 0,46 |
| *Lactobacillus* | 1,16 | -0,24 |
| *Lautropia* | 0,30 | -0,60 |
| *Leptotrichia* | 0,18 | 0,29 |
| *Megasphaera* | -0,17 | -0,14 |
| *Oribacterium* | -0,34 | -0,34 |
| *Prevotella* | -0,24 | -0,25 |
| *Streptococcus* | -0,36 | -0,69 * |
| *Veillonella* | -0,60 * | -0,50 |

**Table S6**. Details of molecular methods

| ***Bacterial strains and growth conditions*** |
| --- |
| The type strains used in this study were *S. mutans* (DSM 20523), *S. sobrinus* (DSM20742), *A. actinomycetemcomitans* (DSM11123) and *Escherichia coli* (ATCC700336). *E. coli* was cultured aerobically in Luria-Bertani medium, *S. mutans* and *S. sobrinus* in blood agar (Oxoid, England), *A.* *actinomycetemcomitans* anaerobically in FAA medium (LAB, England) at 37**°**C. |
| ***DNA extraction*** |
| DNA of type strains was extracted applying QiaAmp DNA mini kit (Qiagen, Hilden, Germany). DNA of saliva was extracted applying QiaAmp Blood Kit (Qiagen, Hilden, Germany) [34] with some modifications.  The saliva (1 ml) was mixed with 4 ml of PBS buffer (pH 7.1), and centrifuged at room temperature (5 min at 3000 x g). The pellet was resuspended in 180 μl PBS. RNase A was added (20 μl of a 20 mg/ml stock solution). After this step, the protocol was followed as described by manufacturer. Pure DNA was eluted from QIAamp spin column with 100 μl of buffer AE, after 5 min. incubation at 70°C. Elution procedure was repeated once (using the collected eluate). Eluted DNA was analyzed on agarose gel, its yield and purity were measured spectrophotometrically. |
| ***Real Time-PCR*** |
| The plasmids containing the amplified region of target bacteria were cloned by using the pGEM-T vector system (Promega, Madison, WI). PCR amplicons of *E. coli*, *S. mutans, S. sobrinus,* and *A. actinomycetemcomitans* were inserted into plasmid vector. The recombinant vector was transformed into chemically competent *E. coli*. We purified plasmids with MaxiPrep (Qiagen) and quantified them by spectrophotometry (Quibit^TM^, Invitrogen) of multiple dilutions [35]. Target DNA was quantified by using serial 10-fold dilutions. Samples and standards were run in triplicates, average values were used for counting bacterial load.  Real-time PCR was carried out using ABI PRISM 7500 HT Sequence Detection System (Applied Biosystems, USA). Amplification reactions were assayed in a total volume of 25 μL containing 2XTaqMan Universal Master mix (PE Applied Biosystems, USA). Each reaction included 2 μL of template DNA, 12.5 pmol of each primer and 3.75 pmol of probe for total bacteria; 20 pmol each primer and 25 pmol probes for *S. mutans* and *S. sobrinus*; 20 pmol of each primer, 10 pmol of TaqMan probe for *A. actinomycetemcomitans* (**Supplementary Table S1**)*.* For detection of total bacteria the thermocycling conditions used were as follows**:** 2 min at 50**°**C and 10 min at 95**°**C followed by 45 cycles consisting of denaturation at 95**°**C for 15 s and annealing-elongation at 60**°**C for 1 min. The negative control was both PCR Master Mixes without DNA. Data were analysed with Sequence Detection Software version 1.6.3 (Applied Biosystem, USA). Samples were run in triplicate, average values were used to calculate bacterial counts. The conversion of Ct (cycle thereshold) of DNA copy number was based on a linear regression equation of DNA standard curve obtained from each assay. |
| ***Microbiome study based on Illumina HiSeq2000 sequencing platform*** |
| Microbial communities were profiled using Illumina® HiSeq 2000 [36]. For this purpose three replicates of PCR products (20 μl of each) were prepared and pooled together. The primers for V6 region of 16s rRNA gene are presented in **Supplementary Table S1** [37,38]. Barcodes for primers were designed with help of Barcrawl program [39] to minimize PCR errors.  Phusion Hot Start High Fidelity Polymerase (Thermo Fisher Scientific) and proposed by its producers reaction mixture was used to carry out PCR reaction. Template concentration in mixture was 0.25-0.5 ng/μl. The following touchdown PCR program was used: denaturation 3 min at 98 ºC, 6 thermal cycles (denaturation at 98 ºC for 5 sec, annealing at 62 ºC for 30 sec with the decrease by 1 ºC every cycle, extension at 72 ºC for 10 sec) followed by 19 different cycles (denaturation at 98 ºC for 5 sec, annealing at 57 ºC with the decrease by 1 ºC every cycle, extension at 72 ºC for 10 sec). The final extension was performed at 72 ºC for 5 min. PCR product concentrations were measured in 2% agarose gel using Quantity One software (Bio-Rad Laboratories). The pooled PCR mixture was cleaned, concentrated (4.5x) with NucleoSpin®Extract II kit (MACHEREY-NAGEL GmbH & Co. KG), and product concentration was measured with Bioanalyzer 2100 (Agilent Technologies). Preparation of paired-end DNA library for sequencing using Illumina® HiSeq 2000 was done with the help of NEXTflexTM PCR-Free DNA Sequencing Kit (BIOO Scientific Corp.). Mock community consisting of seven oral bacterial strains was included together with the saliva samples into NGS pipeline in order to reveal possible contamination and sequencing errors. |
| ***Sequence processing and taxonomic assessment*** |
| Mothur package v.1.13.0 was used to analyse the Illumina sequence data [40] with exception that the clustering step was carried out with external program CROP [41]. The PEAR program was used to assemble the paired-end reads into composite reads [42]. In-house Perl scripts were used to sort the sequences to the samples. The barcodes and primers were then removed. Total initial number of sequences was 29,635,175. Sequences with low quality were discarded. UCHIME was used to remove possible chimeric sequences [43]. In total 29,603,771 usable reads were obtained (total of unique reads 424,121) and from this set the sequences were removed that did not classify as Bacteria. Remaining sequences (17,952,591 sequences – of which 100,460 unique) were clustered with CROP into operational taxonomic units (OTU-s) at 95% similarity level as suggested by [36]. We standardized the sample’s sequences’ number to the lowest sequence number (82830) across the samples only in multivariate analysis. Taxonomic identification of sequences was performed with the RDP Classifier [44] using Greengenes and HOMD 16S rRNA RefSeq Version 11.0 (Human Oral Microbiome Database) with a bootstrap cutoff set at 80%. Richness (i.e. the number) of phylotypes and the Inverted Simpson`s diversity index were calculated. Bray-Curtis measure of similarity was applied to calculate between sample similarity matrix. Sequencing error estimated based on mock community analysis results was 0.01%. |
